# 

**Published:** 1907-12-07

**Authors:** 


The Man with the Broken Ear. By Edmond About. Published at Hatton House, Great Queen Street, W.C. 4d.

To readers of French classics the delightful adventures of the mummy of Colonel Fougas are well enough known, and this excellent translation puts everyone who is unable to follow About's narrative in the original in a position to read the history of the Colonel in English. That the reading will be fraught with much pleasure and amusement we have no doubt. There are few short stories so humorous, so laughable, and so wittily told as this, and whoever wants half an hour's undisturbed enjoyment let him spend fourpence on this little booklet. It is the best value he can get for his money.

### THE GRADUATES' MEDICAL SCHOOLS.

### Diary for the week December 9 to 14.

**NATIONAL HOSPITAL FOR THE PARALYSED AND EPILEPTIC, Queen Square, Bloomsbury, W.C.**

**Lectures at 3.30 p.m.**

December 10, Dr. Batten, **Anterior Poliomyelitis.**

December 13, Dr. Batten, **Paraplegia.**

**THE ROYAL DENTAL HOSPITAL.**

**At 5.30 p.m.**

December 9, Mr. J. G. Turner, **The Ætiology of Palatal Deformities, II.**

**NORTH-EAST LONDON POST-GRADUATE COLLEGE, Prince of Wales's Hospital, Tottenham, N.**

**Surgical, Medical and Special Clinics** daily, Saturdays excepted.

**Lectures at 4.30 p.m.**

December 10, Dr. Basil Price, **Clinical Pathology.**

December 12, Dr. A. G. Auld, **Clinical Medicine.**

**MEDICAL GRADUATES' COLLEGE AND POLYCLINIC, Chenies Street, W.C.**

**Daily** (except Saturday): Clinics, Demonstrations and Practical classes.

**Lectures at 5.15 p.m.**

December 9, Dr. J. Mackenzie, **The Principles of Treatment in Heart Disease.**

December 10, Dr. M. C. Hawkes, **Swedish Medical Gymnastics in Disorders of the Digestive System.**

December 11, Dr. Leonard Guthrie, **Precocious Development.**

December 12, Dr. Radcliffe Crocker, **The Skin Troubles of the Aged.**

**LONDON SCHOOL OF CLINICAL MEDICINE, Seamen's Hospital, Greenwich.**

**Medical and Surgical Clinics.**—Demonstrations and special departments daily.

**Lectures.**

December 9, **2.15 p.m.**, Sir Dyce Duckworth, **Aortic Aneurysms.**

**THE POST-GRADUATE COLLEGE, West London Hospital, Hammersmith, W.**

**Daily at 2:** Medical and Surgical Clinics and $x$-Rays; **at 2.30 p.m.:** Operations.

**Special Departments at 10** (Tuesday, Wednesday, Friday, Saturday), and daily at **2 p.m.**

**Lectures.**

December 9, **12 noon**, Dr. Low, **Pathological Demonstration.**

**At 5 p.m.**

December 9, Dr. Robinson, **Gynæcology.**

December 10, Mr. Armour, **Clinical Lecture I.**

December 11, Mr. Pardoe, **Incontinence of Urine.**

December 12, Mr. Edwards, **Surgical Cases.**

December 13, Mr. Armour, **Clinical Lecture II.**

